# Factors associated with utilization of neoadjuvant chemotherapy in charlson comorbidity zero non-metastatic muscle-invasive bladder cancer patients

**DOI:** 10.1590/S1677-5538.IBJU.2020.0594

**Published:** 2021-02-28

**Authors:** Daniel Au, Eugene K. Lee, Taiye O. Popoola, William P. Parker, Jarron M. Saint Onge, Shellie D. Ellis

**Affiliations:** 1 University of Kansas Health System Department of Urology Kansas CityKS United States Department of Urology, University of Kansas Health System, Kansas City, KS, United States; 2 Health University of Kansas Health System Department of Population Kansas CityKS United States Department of Population, Health University of Kansas Health System, Kansas City, KS, United States

**Keywords:** Urinary Bladder Neoplasm, Cystectomy, Neoadjuvant Therapy

## Abstract

**Background:**

Guideline-based best practice treatment for muscle invasive bladder cancer (MIBC) involves neoadjuvant chemotherapy followed by radical cystectomy (NACRC). Prior studies have shown that a minority of patients receive NACRC and older age and renal function are drivers of non-receipt of NACRC. This study investigates treatment rates and factors associated with not receiving NACRC in MIBC patients with lower comorbidity status most likely to be candidates for NACRC.

**Materials and Methods:**

Retrospective United States National Cancer Database analysis from 2006 to 2015 of MIBC patients with Charlson comorbidity index (CCI) of zero. Analysis of NACRC treatment trends in higher CCI patients was also performed.

**Results:**

15.561 MIBC patients met inclusion criteria. 1.507 (9.7%) received NACRC within 9 months of diagnosis. NACRC increased over time (15.0% in 2015 compared to 3.6% in 2006). Higher NACRC was noted in females, cT3 or cT4 cancer, later year of diagnosis, and academic facility treatment. Lower utilization was noted for blacks and NACRC decreased with increasing age and CCI. Only 16.9% of patients aged 23-62 in the lowest age quartile with muscle invasive bladder cancer and CCI of 0 received NACRC.

**Conclusions:**

Although utilization is increasing, receipt of NACRC remains low even in populations most likely to be candidates. Further study should continue to elucidate barriers to utilization of NACRC.

## INTRODUCTION

Nearly 80.000 people are diagnosed in the United States (U.S.) with bladder cancer annually (1), 15-20% present with muscle-invasive bladder cancer (MIBC) (2). While several international guideline-directed treatment options exist for patients with non-metastatic MIBC, neoadjuvant chemotherapy (NAC) followed by radical cystectomy (RC) is the gold standard (3). Recommendations for neoadjuvant chemotherapy followed by radical cystectomy (NACRC) for MIBC are well-established in the American Urological Association (AUA), U.S. National Comprehensive Cancer Network (NCCN), and European Association of Urology (EUA) guidelines (4, 5). These are based on randomized controlled trials demonstrating improved overall and disease-specific survival (3,4) with cisplatin based neoadjuvant regimens, although a recent non-clinical trial study failed to show survival benefit (6).

Population-based reports demonstrate underutilization of both NAC and RC, 21-27% of MIBC patients ultimately undergo RC (7, 8), far fewer receive NAC prior to surgery (9). While there are clinical reasons patients may not receive neoadjuvant chemotherapy followed by radical cystectomy (NACRC), non-clinical characteristics have been associated with differences in treatment delivery and survival in bladder cancer, including race, age, and gender (10-13). As comorbidity is a strong predictor of overall survival (10), competing causes of mortality may explain observed survival differences. However, it is hypothesized that disparities in utilization of NACRC based on non-clinical characteristics and practice patterns may also play a role (10).

Given the lack of data regarding non-clinical reasons for disparities in NACRC utilization, this study was designed to evaluate factors associated with NACRC utilization in a cohort with lower comorbidity status and therefore most likely to be clinically eligible for NACRC.

## MATERIALS AND METHODS

This study is an Institutional Review Board-exempt 2006-2015 retrospective analysis of the National Cancer Database (NCDB). NCDB is a de-identified, standardized hospital-based registry of United States cancer cases, treatment, and outcomes from over 1.500 facilities across the United States representing 72% of newly diagnosed cancers and 70% of bladder cancers (14).

**Figure 1:**
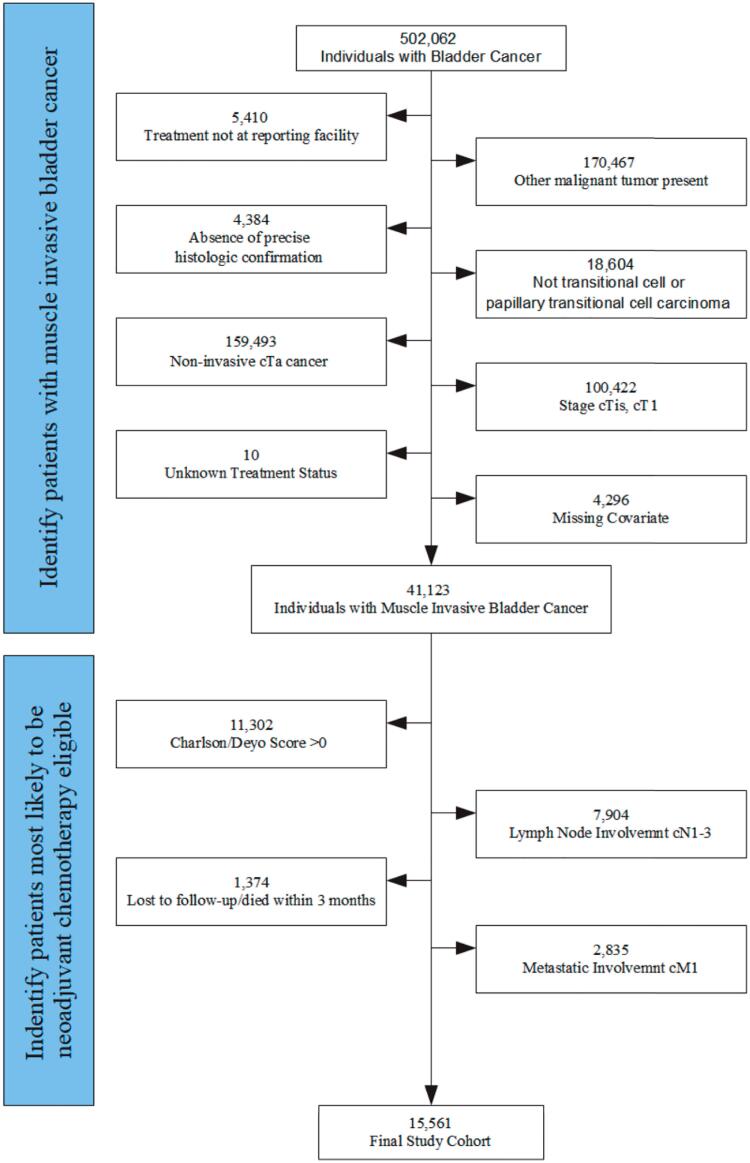
Cohort generation from U.S. National Cancer Database 2006-2015.

Study patients had non-metastatic histologically confirmed transitional cell or papillary transitional carcinoma stages II-III (cT2-4N0). Exclusions included pure or majority squamous cell carcinoma and adenocarcinoma subtypes, secondary malignancies, lack of treatment data and clinical stage lymph node involvement (Figure-1). To attribute the appropriateness of NACRC treatment as best as possible, patients with any of the 15 specific comorbidities measured in the Charlson comorbidity index score (CCI) (15) including renal disease (defined broadly), diabetes, chronic pulmonary disease, myocardial infarction, congestive heart failure, cerebrovascular disease in addition to other conditions which could make a patient unfit for NAC per international guidelines (4, 5) were excluded (Table-1). NCDB CCI of 0 indicates that a patient does not have any record of the 15 CCI comorbidities excluding the cancer diagnosis itself.

**Table 1:** U.S. National Cancer Database (NCDB) Charlson comorbidity index (CCI) criteria and United States Medical Diagnosis IDC-9 Codes (15).

Condition			Charlson Score
Renal Disease			1
	**403.xx, 404.xx**		
		Hypertensive chronic kidney disease stage I-V or end stage renal disease	
	**585.xx**		
		Chronic kidney disease stage I-V or end stage renal disease	
		Chronic kidney disease, unspecified	
	**586.xx**		
		Renal failure unspecified	
	**588.xx**		
		Renal osteodystrophy	
		Nephrogenic diabetes insipidus	
		Secondary hyperparathyroidism	
		Other specified disorders resulting from impaired renal function	
		Unspecified disorder resulting from impaired renal function	
	**V42.0**		
		Renal transplant	
	**V45.1, V56.xx**		
		Renal dialysis	
Cancer (excluded in NCDB CCI calculations)			0
Myocardial Infarction			1
Congestive Heart Failure			1
Peripheral Vascular Disease			1
Cerebrovascular Disease			1
Dementia			1
Chronic Pulmonary Disease			1
Rheumatologic Disease			1
Peptic Ulcer Disease			1
Mild Liver Disease			1
Diabetes			1
Diabetes with Chronic Complications			1
Hemiplegia or Paraplegia			1
Moderate or Severe Liver Disease			1
HIV/AIDS			1

Patients who died or were lost to follow-up within three months of diagnosis were excluded since it would not be possible to measure whether a complete course of chemotherapy was administered. NACRC was defined as cystectomy preceded by at least one course of multi-agent chemotherapy (NAC) both occurring within 9 months of MIBC diagnosis. This time interval was selected to capture patients whose treatment was most likely intended to be delivered primarily rather than as response to a treatment failure.

Although we were able to select for several rounds of multi-agent chemotherapy, the specific multiagent chemotherapy regimen utilized, for example cisplatin based, is not available in NCDB and therefore was not assessed. Single agent chemotherapy was not considered NAC. Cystectomy included “simple/total/complete,” “radical,” “not otherwise specified,” or “pelvic exenteration.” All analyses were controlled for clinicopathologic stage, community, and patient age, gender, race, health insurance, treatment facility type, as well as whether treatment was delivered at more than one facility. Tumor characteristics included clinical T-classification (cT2, cT3, cT4) and grade. To avoid biasing the study by excluding patients with pathologic lymph node involvement found at time of cystectomy pre-cystectomy clinical staging was used. Community factors included rurality, quartile of educational attainment, and income metrics. Facility factors included treatment facility type and U.S. region. Year of diagnosis was analyzed for trends.

Given concern for internal validity of CCI score in NCDB since only 11.302 patients were excluded for CCI greater than 0 from the initial 41.123 MIBC patients meeting cohort identification criteria, a sensitivity analysis to assess rates of NACRC in higher CCI patients was also performed. These patients represented those excluded from the main cohort for co-morbidity alone and otherwise meeting all other study inclusion and exclusion criteria.

All variables were considered categorical and summarized with frequencies and percentages with comparisons between NACRC and non-NACRC using Chi-squared tests. To assess for independent associations of factors and control for clustering of patients within facility a mixed effects logistic regression modeling was performed (16). Models were summarized using adjusted odds ratios (OR) and 95% confidence intervals with 2-sided p-values <0.05.

## RESULTS

**Figure 2:**
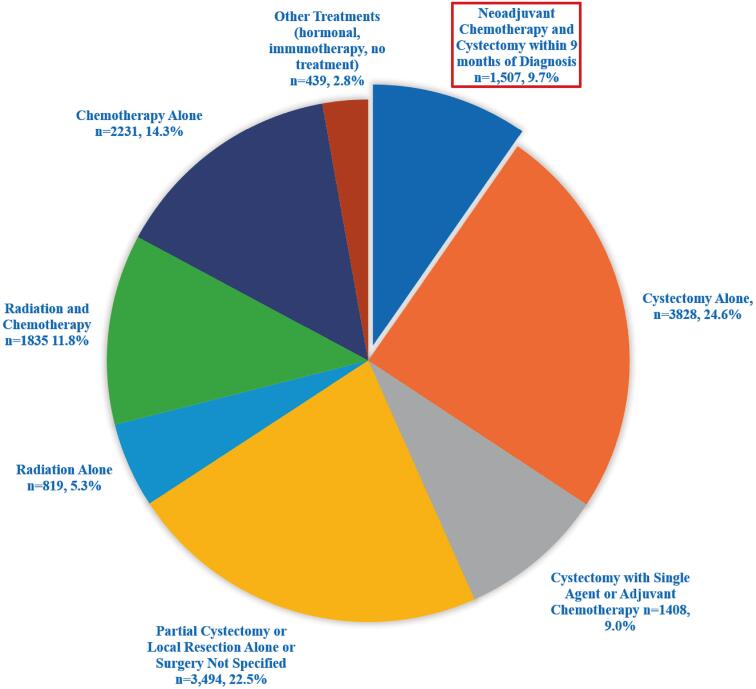
Rates of treatment utilization 2006-2015 in U.S National Cancer Databe non-matastatic muscle invasive baldder cancer patients with Charlson comorbidity 0.

Among the 502.062 patients with bladder cancer identified, 41.123 had MIBC and no secondary malignancy. All treatment and data inclusion criteria including CCI score of 0 was met for 15.561 patients (Figure-1). NACRC was utilized in 9.7% (n=1.507), while 24.6% (n=3.828) received cystectomy alone, 9.0% (n=1.408) cystectomy with single agent or adjuvant chemotherapy, 22.5% (n=3.494) non-cystectomy surgical treatments such as partial cystectomy or local resection, 17.1% (n=2.654) radiation with or without chemotherapy, 14.3% (n=2.231) chemotherapy alone, and 2.8% (n=439) hormonal, immune, or no treatment (Figure-2). Overall, the large majority of patients, 90.3% (n=14.054), did not receive treatment that qualified as NACRC. Only 43.3% (n=6.743) received a complete cystectomy as part of their management.

NACRC use increased from 3.6% in 2006 to 15.0% in 2015 among non-metastatic MIBC patients with CCI 0 (Table-2). Only 16.9% of patients aged 23-62 in the lowest age quartile with muscle invasive bladder cancer and CCI of 0 received NACRC (Table-2) increasing over time to 26.1% in 2015. Significant differences in all clinical factors were observed between patients who did and did not receive NACRC therapy in unadjusted analysis with the exception of rurality of patient residence (Table-2). In adjusted multivariable analysis (Table-3), female gender, later year of diagnosis, and having either cT3 or cT4 were significantly associated with greater odds of receiving NACRC (p <0.001, p <0.001, p <0.01 respectively). In contrast, older patient age, black race, unknown ethnicity, and being treated at a non-academic/research program facility were associated with lower odds of receiving NACRC (p <0.001, p=0.03, p=0.05, p <0.001 respectively).

**Table 2:** Characteristics by receipt NACRC for patients with MIBC and Charlson Comorbidity Index 0.

Characteristics		Overall	Did not receive NAC + RC	Received NAC + RC	p-value
		*N*	*N (%)*	*N (%)*	
**Overall**		15,561	14,054 (90.3)	1,507 (9.7)	
**Age categorized by quartiles**					<0.001
	23-62	3,820	3,174 (83.1)	646 (16.9)	
	63-71	4,188	3,628 (86.6)	560 (13.4)	
	72-81	3,782	3,527 (93.3)	255 (6.7)	
	82-90	3,771	3,725 (98.8)	46 (1.2)	
**Gender**					0.003
	Female	4,778	4,264 (89.2)	514 (10.8)	
	Male	10,783	9,790 (90.8)	993 (9.2)	
**Race**					0.02
	White	14,138	12,738 (90.1)	1,400 (9.9)	
	Black	1,030	948 (92.0)	82 (8.0)	
	Asian/Native Hawaiians/Pacific Islanders or Other*	393	368 (93.6)	25 (6.4)	
**Ethnicity**					<0.001
	Non-Hispanic	14,635	13,182 (90.1)	1,453 (9.9)	
	Hispanic	178	160 (89.9)	18 (10.1)	
	Unknown	748	712 (95.2)	36 (4.8)	
**Insurance status**					<0.001
	Uninsured	511	452 (88.5)	59 (11.5)	
	Private Insurance	4,630	3,947 (85.3)	683 (14.8)	
	Medicaid	635	545 (85.8)	90 (14.2)	
	Medicare	9,324	8,698 (93.3)	626 (6.7)	
	Other Government or Insurance Status Unknown*	461	415 (89.4)	49 (10.6)	
**Proportion of adults who did not graduate from high school categorized as quartiles**					<0.01
	≥ 29%	2,124	1,957 (92.1)	167 (7.9)	
	20% - 28.9%	3,754	3,400 (90.6)	354 (9.4)	
	14% - 19.9%	4,022	3,624 (90.1)	398 (9.9)	
	<14%	5,661	5,073 (89.6)	588 (10.4)	
**Median household income categorized as quartiles**					0.02
	<$30,000	1,765	1,612 (91.3)	153 (8.7)	
	$30,000 - $35,999	2,824	2,581 (91.4)	243 (8.6)	
	$36,000 - $45,999	4,541	4,102 (90.3)	439 (9.7)	
	$46,000 +	6,431	5,759 (89.6)	672 (10.5)	
**Rurality of Patient's Residence**					0.98
	Non-Rural	14,912	13,475 (90.4)	1,437 (9.6)	
	Rural	292	264 (90.4)	28 (9.6)	
**Year of diagnosis**					<0.001
	2006	1,070	1,031 (96.4)	39 (3.6)	
	2007	1,259	1,194 (94.8)	65 (5.2)	
	2008	1,566	1,474 (94.1)	92 (5.9)	
	2009	1,797	1,670 (92.9)	127 (7.1)	
	2010	1,452	1,325 (91.3)	127 (8.7)	
	2011	1,500	1,373 (91.5)	127 (8.5)	
	2012	1,581	1,405 (88.9)	176 (11.1)	
	2013	1,668	1,455 (87.2)	213 (12.8)	
	2014	1,806	1,545 (85.6)	261 (14.5)	
	2015	1,862	1,582 (85.0)	280 (15.0)	
**Facility type**					<0.001
	Academic/Research Program	5,783	4,923 (85.1)	860 (14.9)	
	Community Cancer Program	1,630	1,545 (94.8)	85 (5.2)	
	Comprehensive Community Cancer Program	6,190	5,790 (93.5)	400 (6.5)	
	Integrated Network Cancer Program or Other type of cancer program*	1,958	1,796 (91.7)	162 (8.3)	
**Facility location**					<0.001
	New England	1,078	954 (88.5)	124 (11.5)	
	Middle Atlantic	2,494	2,272 (91.1)	222 (8.9)	
	South Atlantic	3,395	3,083 (90.8)	312 (9.2)	
	East North Central	2,795	2,505 (89.6)	290 (10.4)	
	East South Central	1,007	920 (91.4)	87 (8.6)	
	West North Central	1,263	1,100 (87.1)	163 (12.9)	
	West South Central	990	920 (92.9)	70 (7.1)	
	Mountain	733	656 (89.5)	77 (10.5)	
	Pacific	1,806	1,644 (91.0)	162 (9.0)	
**Multiple source of records**					<0.001
	Records submitted by only one facility	11,763	10,829 (92.1)	934 (7.9)	
	Records submitted by more than one facility	3,798	3,225 (84.9)	573 (15.1)	
**Clinical T-classification**					<0.001
	2	12,479	11,367 (91.1)	1,112 (8.9)	
	3	1,556	1,327 (85.3)	229 (14.7)	
	4	1,526	1,360 (89.1)	166 (10.9)	
**Grade**					<0.001
	Well differentiated or Moderately and moderately-well differentiated*	718	677 (94.3)	41 (5.7)	
	Poorly differentiated	6,442	5,943 (92.3)	499 (7.8)	
	Undifferentiated, anaplastic	6,505	5,752 (88.4)	753 (11.6)	
	Cell type not determined, not stated or not applicable	1,896	1,682 (88.7)	214 (11.3)	

Significance determined by Pearson Chi-square

Rows may equal more than 100% due to rounding

* Combined to protect confidentiality

**Table 3:** Associations of clinical features with receipt of NAC for patients with MIBC and Charlson Comorbidity Index 0.

Variable		OR	95% CI	p-value
**Age categorized by quartiles**				
	23-62	Referent		
	63-71	0.76	0.64- 0.89	0.001
	72-81	0.39	0.32-0.48	<0.001
	82-90	0.07	0.05-0.10	<0.001
**Gender**				
	Male	Referent		
	Female	1.46	1.28-1.66	<0.001
**Race**				
	White	Referent		
	Black	0.67	0.52-0.88	0.003
	Asian/Native Hawaiians/Pacific Islanders	0.65	0.38-1.11	0.11
	Other	0.41	0.17-0.99	0.05
**Ethnicity**				
	Non-Hispanic	Referent		
	Hispanic	1.15	0.66-2.03	0.62
	Unknown	0.70	0.47-1.04	0.08
**Insurance status**				
	Private Insurance	Referent		
	Uninsured	0.95	0.69-1.30	0.75
	Medicaid	0.93	0.71-1.23	0.62
	Medicare	0.85	0.72-0.99	0.05
	Other Government	1.27	0.80-2.00	0.31
	Insurance Status Unknown	1.04	0.62-1.73	0.14
**Number of adults who did not graduate from high school, quartiles**				
	≥ 29%	Referent		
	20% - 28.9%	1.21	0.96-1.53	0.11
	14% - 19.9%	1.17	0.91-1.51	0.22
	<14%	1.19	0.91-1.56	0.20
**Quartile of median household income**				
	<$30,000	Referent		
	$30,000 - $35,999	0.85	0.66-1.09	0.20
	$36,000 - $45,999	0.90	0.70-1.15	0.40
	$46,000 +	1.00	0.76-1.32	0.99
	Rurality of Patient's Residence	0.80	0.51-1.25	0.33
**Year of diagnosis**				
	2006	Referent		
	2007	1.24	0.80-1.93	0.33
	2008	1.71	1.14-2.58	0.01
	2009	1.99	1.34-2.94	<0.001
	2010	2.54	1.71-3.76	<0.001
	2011	2.40	1.61-3.56	<0.001
	2012	3.42	2.33-5.03	<0.001
	2013	3.69	2.52-5.40	<0.001
	2014	4.61	3.17-6.72	<0.001
	2015	4.89	3.36-7.12	<0.001
**Facility type**				
	Academic/Research Program	Referent		
	Community Cancer Program	0.40	0.30-0.54	<0.001
	Comprehensive Community Cancer Program	0.54	0.44-0.66	<0.001
	Integrated Network Cancer Program	0.61	0.46-0.80	<0.001
**Facility location**				
	New England	Referent		
	Middle Atlantic	0.58	0.39-0.86	<0.01
	South Atlantic	0.68	0.47-0.99	<0.05
	East North Central	0.85	0.59-1.22	0.38
	East South Central	0.75	0.47-1.20	0.23
	West North Central	1.21	0.79-1.84	0.39
	West South Central	0.42	0.26-0.69	<0.01
	Mountain	0.98	0.61-1.57	0.92
	Pacific	0.62	0.41-0.93	0.02
**Multiple source of records**				
	Records submitted by only one facility	Referent		
	Records submitted by more than one facility	1.51	1.33-1.72	<0.001
**Stage**				
	2	Referent		
	3	1.74	1.46-2.08	<0.001
	4	1.34	1.10-1.64	0.003
**Grade**				
	Referent		
	Moderately or moderately well differentiated,	1.37	0.63-3.00	0.43
	Poorly differentiated	1.63	0.82-3.23	0.16
	Undifferentiated, anaplastic	1.89	0.95-3.75	0.07
	Cell type not determined	1.72	0.86-3.47	0.13
	Constant	0.04	0.02-0.10	<0.001
	Facility ID Var (_cons)	0.54	0.40-0.71	

All odds ratios in table modeled by multi-level mixed effects logistic regression

Over time, between the first half of the study period 2006-2010 (4th quartile age OR 0.05, p <0.001) and the second half 2011-2015 (4th quartile age OR 0.06, p <0.001) trends in odds of receiving NACRC in older patients compared to the youngest patients did not change. Black race was still associated in later years 2011-2015 (OR 0.68, p=0.02) with lower odds of NACRC compared to white race but this was higher than the first half of the study period (OR 0.58 p=0.02). Study patient's rates of receiving NACRC increased at academic facilities and community programs from 2006-2010 (11.2% academic, 3.4% community, p <0.00) versus 2011-2015 (17.7% academic, 8.8% community, p <0.00). Using academic centers as referent, community cancer programs had increased odds of providing NACRC from 2011-2015 (OR 0.44, p <0.001) compared to 2006-2010 (OR 0.34, p <0.001) but this remained below academic centers. The increased rates of NACRC at community care facilities was proportionally less than the increased rates at integrated cancer programs from 2011-2015 (OR 0.67, p=0.01) versus 2006-2010 (OR 0.50, p <0.001). Integrated cancer programs are in-between community and academic facilities in terms of facility medical resources. Rates of NACRC at integrated cancer programs also remained below academic centers. Female gender odds of NACRC receipt did not change over the study course. There was no statistically significant association with insurance status/type, level of education and household income, or tumor grade throughout the study.

In sensitivity analysis of higher CCI patients while maintaining all other study criteria, higher CCI score was associated with lower rates of NACRC. Rates of NACRC were 7.3% for CCI 1, 5.7% for CCI 2, and 4.4% for CCI 3 or more (Table-4a). The odds ratio of receipt of NACRC compared to CCI 0 patients was 0.77 (95% CI 0.69-0.86) for CCI 1, 0.58 (95% CI 0.48-0.71) for CCI 2, and 0.45 (95% CI 0.32-0.64) for CCI 3 or more (Table-4b).

**Table 4a:** Rates NACRC for patients with MIBC by Charlson Comorbidity Index score 2006-2015.

		Overall	Did not receive NAC + RC	Received -NAC + RC	p-value
		N	N (%)	N (%)	<0.001
**Overall**		24,157	22,072 (91.3)	2,085 (8.6)	
Charlson Comorbidity Index*					
	0	15,561	14,054 (90.3)	1,507 (9.7)	
	1	5,951	5,514 (92.7)	437 (7.3)	
	2	1,902	1,794 (94.3)	108 (5.7)	
	3+	743	710 (95.6)	33 (4.4)	

**Table 4b:** Odds ratios receipt of NACRC for patients with MIBC all Charlson Comorbidity Scores.

Variable		OR	95% CI	p-value
Charlson Comorbidity Index*				
	0	Referent		
	1	0.77	0.69-0.86	<0.001
	2	0.58	0.48-0.71	<0.001
	3+	0.45	0.32-0.64	<0.001

* Significance determined by Pearson Chi-square

Modeled by multi-level mixed effects logistic regression

Rows may equal more than 100% due to rounding

## DISCUSSION

Overall, 9.7% of patients with non-metastatic MIBC in patients with a CCI of zero received multiagent NACRC within nine months of diagnosis. Although NACRC use increased over time, consistent with other reports of NAC utilization (9). NACRC rates among lower CCI patients, those recommended by European and U.S. guidelines to receive NACRC, remain remarkably low reaching only 15% in 2015. Study observed rates of NACRC are not an artifact of delay of care, few patients received NACRC outside of a 9-months from diagnosis treatment window (0.3%).

Among CCI 0 patients with MIBC, 16.9% of patients aged 23-62 in the lowest age quartile received NACRC. The receipt of NACRC decreased with increasing age with only 1.2% of the oldest quartile receiving NACRC therapy (Table-2). This corresponds to an adjusted odds ratio of oldest quartile compared to youngest quartile for receipt of NACRC of 0.07 (95% CI 1.28-1.66) (Table-3). The remarkably low rates of NACRC in younger patients with CCI of 0 in particular is likely to be inappropriate and concerning for several reasons.

First, given association of increasing comorbidity with aging (17, 18), low NACRC rates are especially alarming in younger patients with the lowest CCI scores who constitute the patient population in NCDB most likely to be eligible for NACRC. Younger patients in general are least likely to have renal impairment or other contraindications to NACRC. Comorbid contraindications to NACRC, especially renal insufficiency, are more likely to be present in older patients (19). Secondly, younger patients are those most likely to live long enough to accrue the small incremental gain in overall and cancer specific survival from NACRC which was around 5-8% at five years post-treatment in the original trials (3, 4). Older patients have shorter residual life expectancy and may be less likely to appreciate incremental survival gains.

Notably, a significant portion of patients in this cohort appeared to receive off guideline therapies such as hormonal/immune therapy treatments only, cystectomy with single agent chemotherapy, and local resection or chemotherapy alone (Figure-2). A significant portion of study patients also underwent alternative bladder preservation therapies trimodal therapy with chemo, radiation and maximal TURBT and partial cystectomy (Figure-2), over the study period combined these modalities accounted for more than triple those who underwent NACRC. While treatment with bladder preserving therapies are emerging modalities with promise (20) (particularly in limited T2 disease and those with significant comorbidity), they are not considered standard treatment especially in a healthier patient cohort such as this study.

Apart from age and recency of diagnosis, this study found that gender, type of treatment facility, and black race were associated with the receipt of NACRC on adjusted analysis of CCI 0 patients. Female patients with non-metastatic MIBC and CCI 0 were more likely to receive NACRC than male patients OR 1.46 (95% CI 1.28-1.66) (Table-3). This contrasts to some prior studies (21, 22) which have found women in general were more likely to receive substandard and delayed care then men across the bladder cancer disease spectrum. These prior studies did not look specifically at NACRC in CCI 0 patients and generally studied a significantly earlier time period. Black race was associated with lower NACRC utilization (Table-3). Systematic differences in care patterns by race may explain differences disease-specific survival (23). NCDB does not proportionally reflect U.S. racial groups and therefore racial disparity findings should be approached cautiously.

Given the long timescale of this study starting in 2006 when NAC recommendations were more recent, analysis of the first five years 2006-2010 compared to the last five years of the study period 2011-2015 was also performed to assess the nature of these. Between these two periods overall rates of NACRC increased 5.8% versus 12.6%, while still low this does represent implementation of recommendations over time. Over this time, black patients did become more likely to receive NACRC but were still not at likely as Caucasian patients to receive it at the end of the study period. When comparing the early study period to later, academic centers experienced greater NACRC implementation than community centers and integrated cancer centers (facilities that are functionally in-between academic and community facilities in terms of level or resources). This likely represents improved implementation of guidelines at academic centers who are more specialized with higher volumes than community care centers.

Overall, during the study patients treated at academic/teaching facilities were twice as likely to receive NACRC for non-metastatic MIBC. This adds to previous reports that care regionalized to high volume centers of excellence leads to decreased morbidity, mortality, decreased length of stay, and decreased readmissions (24-26). The underlying reason for this disparity in care between facility types remains unknown. Theoretically, academic centers may be more likely to have the resources and skills to perform RC, coordinate with medical oncologists to ensure appropriate and timely delivery of NAC, and possess the referral relationships to ensure high quality care. However, future research is required to better understand these differences in care utilization (27) and how to facilitate appropriate referral to regional centers when, by virtue of lack of resources, NACRC is not feasible. In addition, patient's willingness to seek care far from home is not well explored in the literature and underscores the lack of data describing patient's preferences in obtaining NACRC treatment for their disease.

Due to the multidisciplinary nature of NACRC requiring medical oncology, urology, social work, and nutrition expertise (28) to deliver a unified care pathway, barriers may also vary by provider type. For example, NACRC requires urologists hold their portion of care and collaborate with oncology which may carry potent organizational and coordination of care barriers. Future quality improvement and implementation science-based studies in this area may be a useful in better dissemination of NACRC guidelines and identifying and mitigating treatment center type and provider type barriers to NACRC.

Both clinical and non-clinical factors preclude patients from receiving NAC. In this study, some non-clinical factors that may preclude patients from starting or completing a NACRC treatment regimen were attempted to be isolated in order to examine them separately. There are racial and gender care disparities that may be unique to the United States, however disparities in guideline implementation by facility type is likely to be applicable to every health system. NACRC is a complex treatment modality that requires care coordination between multiple physician and non-physician medical specialty types. Even after initiating a NACRC treatment plan, patients, their families, and medical providers encounter numerous obstacles to completing the combined medical and surgical regimen. These pitfalls are all the more difficult to surmount at facilities without the resources of a centralized high-volume experienced facility.

Compared to other recent studies already cited, this report is unique in that it selected for patients without documented CCI in order to only include patients with highest likelihood of NACRC eligibility. This study is not without limitations however and to fully interpret the results, it is important to understand CCI and other limitations within NCDB.

Screening patients in this study by CCI only allows for the exclusion of patients on the basis of any one of 15 categories of comorbidity. This includes several important categories that may preclude eligibility for NACRC including any history of renal disease, diabetes, chronic pulmonary disease, myocardial infarction, congestive heart failure, and cerebrovascular disease. Renal disease may be the most relevant of the CCI conditions in terms of NAC eligibility. Careful evaluation of the specific Charlson definition of renal disease reveals a broadly inclusive definition including all stages, I (mild) though V (severe), renal impairment (Table-1). It is important to note however that CCI does not cover all comorbidity types. Patients with a score of 0 could still have comorbidity if they are not conditions specifically included in CCI (15). CCI in particular does not cover all comorbidity relevant for clinical decision making for NAC or RC appropriateness. For example, other important measures such as creatinine clearance, myelosuppression, hearing loss, and Eastern Cooperative Oncology Group (ECOG) status are missing in this analysis. This significant study limitation may contribute to the lower observed NACRC utilization rates in this study if these unmeasured by CCI comorbidities made patients unfit for NACRC.

Given concern for the accuracy of utilizing CCI as a proxy for likeliness of NAC eligibly, a sensitivity analysis of higher CCI patients otherwise meeting study criteria was performed. This did confirm patients with higher CCI had with lower odds ratio of receipt of NACRC as would be expected if NCBD CCI data had internal validity. Additionally, prior studies have evaluated the internal validity of CCI within NCDB (29). One study found NCDB CCI scores compared to a chart review-based CCI calculation underestimated comorbidity for 19.1-36.2% of patients (30). Adherence to NACRC may be underestimated if, due to imperfect capture of comorbidity, this unknowingly included patients with significant comorbidity.

Another area of study limitation is the imperfect granularity of variant bladder histology within the NCDB. This study included only muscle invasive bladder cancers identified as transitional cell carcinoma or papillary transitional cell carcinoma. We may have included patients with a squamous cell component as long as it was a minority of the sample because these are coded within NCDB as TCC, we are unable to know how many patients fit into this category due to database limitations. Pure or majority squamous cell carcinoma and adenocarcinoma/signet ring cell sub-types are coded within the NCDB database and were specifically excluded. NCDB does not codify all subtypes in the database such as micropapillary, nested, plasmacytoid, or sarcomatoid which may be poorly suited for NAC and these may have been unknowingly included in the analysis.

This study is also not able to determine what specific multi-agent chemotherapy regimen (for example MVAC - methotrexate, vinblastine, doxorubicin, cisplatin versus GS - Gemcitabine Cisplatin) a patient received because this data is not available within NCDB. Not all multi-agent chemotherapy would be standard of care, for example carboplatin-based regimens are specifically not recommended per guidelines (5) and if given can be more harmful than beneficial. Given NACRC was defined in this study as cystectomy preceded by at least one course of multi-agent chemotherapy (NAC), it is possible that non-standard of care regimens were included in the analysis as standard of care. If this is present in our data, this would actually result in an overestimation in the rates of guideline appropriate NACRC however and true guideline adherence rates would be even lower than are reported in this study.

## CONCLUSION

Only 9.7% of all patients and 16.9% of the youngest study quartile patients aged 23-62 with non-metastatic muscle invasive bladder cancer and low Charlson comorbidity received likely best practices treatment with timely multi-agent neoadjuvant chemotherapy followed by cystectomy. Utilization of neoadjuvant chemotherapy followed by cystectomy did increase over time. Identifiable factors associated with higher utilization were female gender, cT3 or cT4 cancer, later year of diagnosis, and academic or research facility treatment. Lower utilization was noted for blacks and NACRC utilization decreased with increasing age and CCI score. Academic treatment facilities exhibited a greater odds of NACRC treatment utilization than community centers over time. Community practice settings continue to lag further behind in NACRC application.

Although utilization of NACRC is increasing over time, rates remain low even in populations most likely to be healthy enough to be treatment candidates. Rates varied but also remained low overall across treatment facility types. This data highlights the need for further quality improvement and implementation science studies to elucidate barriers to adoption of the complex care coordination treatment of muscle invasive bladder cancer with neoadjuvant chemotherapy followed by radical cystectomy.

## ABBREVIATIONS

MIBC: Muscle Invasive Bladder Cancer
NAC: Neoadjuvant Chemotherapy
RC: Radical Cystectomy
NACRC: Neoadjuvant Chemotherapy followed by Radical Cystectomy
CCI: Charlson Comorbidity Index Score
